# Evaluating Heavy Metal Stress Levels in Rice Based on Remote Sensing Phenology

**DOI:** 10.3390/s18030860

**Published:** 2018-03-14

**Authors:** Tianjiao Liu, Xiangnan Liu, Meiling Liu, Ling Wu

**Affiliations:** School of Information Engineering, China University of Geosciences, Beijing 100083, China; 3004160002@cugb.edu.cn (T.L.); liuml@cugb.edu.cn (M.L.); wuling@cugb.edu.cn (L.W.)

**Keywords:** remote sensing phenology, heavy metal stress, rice, WRT, time-series

## Abstract

Heavy metal pollution of croplands is a major environmental problem worldwide. Methods for accurately and quickly monitoring heavy metal stress have important practical significance. Many studies have explored heavy metal stress in rice in relation to physiological function or physiological factors, but few studies have considered phenology, which can be sensitive to heavy metal stress. In this study, we used an integrated Normalized Difference Vegetation Index (NDVI) time-series image set to extract remote sensing phenology. A phenological indicator relatively sensitive to heavy metal stress was chosen from the obtained phenological periods and phenological parameters. The Dry Weight of Roots (WRT), which directly affected by heavy metal stress, was simulated by the World Food Study (WOFOST) model; then, a feature space based on the phenological indicator and WRT was established for monitoring heavy metal stress. The results indicated that the feature space can distinguish the heavy metal stress levels in rice, with accuracy greater than 95% for distinguishing the severe stress level. This finding provides scientific evidence for combining rice phenology and physiological characteristics in time and space, and the method is useful to monitor heavy metal stress in rice.

## 1. Introduction

Heavy metal pollution in farmland has become an increasingly serious problem for modern agriculture that urgently requires a solution. Heavy metal pollution can cause crop growth stress, affecting the yield and quality of crops as well as severely affecting human health after entering the human body through the food chain [1,2]. Polluted land now accounts for one-sixth of China’s total arable land. Only by understanding the distribution and severity of the pollution can remediation measures be more targeted. Therefore, accurately monitoring heavy metal stress in real time is of great importance [3,4,5]. Compared to traditional ground measurement methods, remote sensing (RS) technology is a more effective method for studying heavy metal stress in farmland. The advantages of RS include providing real-time measurement with continuity, high efficiency and non-destruction, and allowing large-scale observation [6,7,8].

As the change in a plant’s physiological elements, such as chlorophyll content, cell structure, and water or nitrogen content, can be monitored by the reflection spectrum, researchers have identified the relationship between sensitive spectral characteristics and heavy metal concentrations or physiological factors by establishing empirical or semi-empirical models. However, heavy metal stress information has not been acquired throughout the entire growth stage, since the spectral data only reflected the stress state of one or several growth stages, which may lead to randomness. Research on the physiological and ecological effects of heavy metal pollution in rice demonstrated that heavy metal poisoning can lead to thin plant growth, short leaves, serious yellowing, and phenological changes [9,10,11,12]. Phenology is often used as an important indicator of crop yield estimation and field management; the length of the phenological period and growth rate are important factors for crop growth simulation [12,13,14,15,16]. The phenological information extracted using remote sensing technology not only reflects the continuous growth and stress state of rice throughout the entire growth stage, but also avoids uncertainty of selecting the spectral parameters most sensitive to physiological elements. Furthermore, researchers can draw inferences about the condition of crops and their environment (e.g., soil factor) from the information on the timing and progression of crop development [17], and heavy metals as a soil factor effects crop growth [7], therefore, exploring the relationship between rice phenology and heavy metal stress helps to understand the response mechanism of rice to the concentration of heavy metals in the soil [15]. In previous studies, Vegetation Indexes (VIs) with distinct seasonal rhythm were commonly used in phenology. The Normalized Difference Vegetation Index (NDVI) reflects the seasonal variation in the characteristics of rice to accurately reveal the dynamic state of rice according to the spectral reflectivity characteristics of vegetation in the visible and near infrared bands [18].

Phenology has many important applications for crop identification, production estimation, and growth monitoring [19,20,21,22,23]. The rapid development of remote sensing phenology monitoring has become a bridge between phenology and other disciplines, and increasing amounts of satellite data are being applied in phenology [24,25,26,27,28,29,30]. Compared with Advanced Very High-Resolution Radiometer (AVHRR) data, moderate resolution imaging spectroradiometer (MODIS) data covers a wider range of spectral bands with higher spatial and temporal resolution. However, for small-scale crops, the low spatial resolution of MODIS data is unable to achieve accurate recognition. The low temporal resolution in SPOT and Landsat cannot indicate the crop growth period. The Charge-Coupled Device (CCD) sensors on board China’s Environment Satellites are superior as they provide appropriate temporal and spatial resolution and are open to public, making the data suitable for phenological research. In addition, the rainy season during the growing season of rice in the southern region of China, results in insufficient images, which lower the accuracy of phenological information acquisition. To monitor heavy metal stress levels in rice with phenology, collecting more images is essential for extracting accurate phenological information.

In previous phenology research, the seasonal changes in the belowground parts (roots), and the differences in the root phenology from the phenology of the aboveground parts, were often neglected. Hence, we extended the phenology research from the aboveground plant to the belowground parts. The root system of rice is mainly developed during the vegetative growth stage. Heavy metal toxicity first affects the growth of crop roots, as roots are more sensitive to heavy metal stress compared to stems, leaves, and storage organs. Heavy metal pollution inhibits root growth and results in the decrease in root weight [31,32,33]. Therefore, timely monitoring heavy metal stress in rice can be achieved by measuring the root weight. However, the impact of heavy metal stress on the root decreases during reproductive growth stage. Meanwhile, the decrease in dry matter accumulation and distribution rate, caused by the decrease in photosynthesis intensity under heavy metal stress, leads to a phenology change in rice [31]. Therefore monitoring phenology compensates for the deficiency in reduced sensitivity to heavy metal stress as roots age, to further explore the effects of heavy metal stress in the middle and later stages of rice growth. The World Food Study (WOFOST) model involves phenology, dry weight accumulation, and physiological function, can be used to obtain the Dry Weight of Roots (WRT) values [7,34,35]. The WRT obtained based on phenology can more accurately describe the seasonal changes in root weight. In this study, phenology and WRT were combined to improve the accuracy of heavy metal stress monitoring in rice. Our results will provide a resource for researchers and agricultural managers to monitor heavy metal stress in rice efficiently.

## 2. Study Area and Materials

### 2.1. Study Area

The study area, ranging between 26°03′–28°01′ N and 112°57′–114°07′ E, is located in the Zhuzhou area, downstream from Xiangjiang in Hunan Province. The climate is categorized as subtropical monsoon humid with four distinct seasons, abundant rainfall, adequate light and heat. The soil is mostly red, with high organic matter content. These climate and soil conditions promote high yields of rice, which is an important grain commodity in the area. The Xiangjiang River is the source of domestic, industrial, and agricultural water in Hunan. The long-term discharge of large quantities of industrial wastewater, gases, and residues has contaminated water, soil, and crops in the Xiangjiang watershed with different levels of heavy metals. Many rice fields adjacent to the Xiangjiang watershed in Zhuzhou have become seriously polluted.

**Figure 1:**
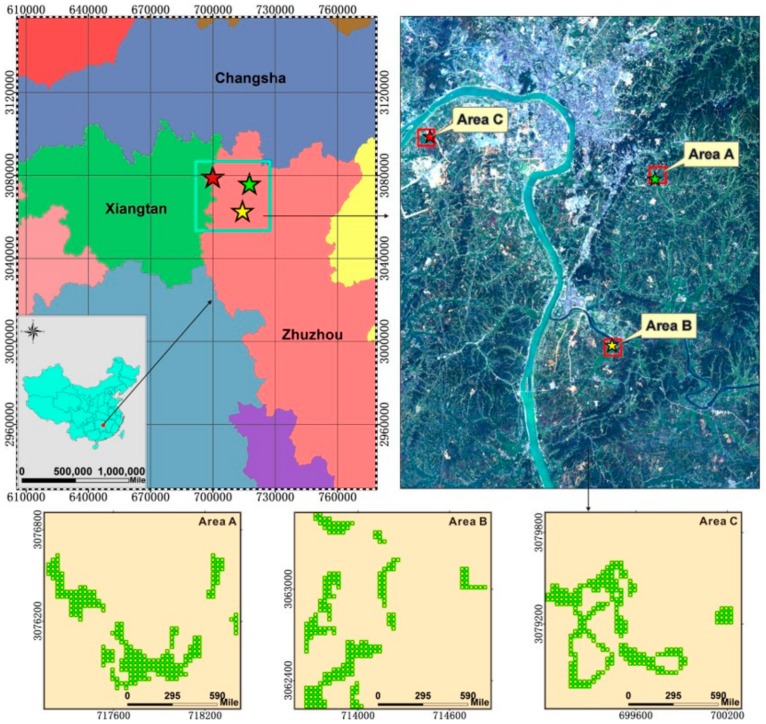
The geographical location of the study area in Hunan Province, China, and the spatial distribution of sample points.

According to the meteorological data provided by the meteorological station in Zhuzhou, the annual average temperature and precipitation in the study area were 16–18 °C and 1257 mm, respectively. The total number of sunshine hours during the rice growing period was 969.6 h, and the degree of suitability of the temperature, illumination, precipitation, and synthesis were all above 0.7 [36,37]. In the study area, we selected three 1.28 km × 1.28 km rice fields labeled as A, B, and C (Figure 1). They have similar climatic condition and vary in heavy metal stress level. The main type of rice in that area is Boyou 9083. The soil environmental quality standard is used to evaluate the pollution levels [38]. The pollution levels for areas A, B and C are categorized as “mild level”, “moderate level”, and “severe level”, respectively (Table 1). In addition, intensive cultivation patterns were used in all experimental areas to ensure adequate irrigation and sufficient fertilizers in paddy fields without pests, weeds, or other environmental issues.

### 2.2. Data Collection

The datasets collected included remote sensing data, meteorological data, and crop data from June to September in 2013, covering the entire rice growing season in Zhuzhou. Three types of remote sensing images were used: CCD images of HJ-1A/B, Operational Land Imager (OLI) images of Landsat-8, and Enhanced Thematic Mapper Plus (ETM+) images of Landsat-7. Comparing the sensor specifications of the three satellites (Table 2), the spatial resolution was 30 meters for all the images, and the band range was the same in the visible and near infrared bands. In this study, 30 CCD images, three OLI images, and five ETM+ images were selected for the remote sensing phenology. According to the absolute radiation calibration coefficient of HJ-1A/B, released by the China Resources Satellite Application Center in 2013, radiometric calibration and layer stacking were applied to the CCD images. Radiometric calibration and atmospheric correction were also needed for both ETM+ and OLI images. The Fast Line-of-sight Atmospheric Analysis of Spectral Hypercubes (FLAASH) model was used for atmospheric correction in the three types of remote sensing images, and geometric correction was based on CCD images. The corrected root-mean-square error (RMSE) was less than 0.5 pixels. In addition, due to the failure of the Landsat-7 airborne scan line corrector (SLC) in May 2003, gapfill processing was required for the ETM+ images.

Daily meteorological data for the whole year, including temperature, daylight, precipitation, and solar radiation, was provided by the Zhuzhou Weather Station. The temperature, daylight, and precipitation data were used to evaluate the climate in the three paddy fields. The temperature and solar radiation data served as important input parameters for the WOFOST model. The measured crop data mainly included Leaf Area Index (LAI), and the heavy metal content of soil and rice. In each study area, 20–30 30 m × 30 m-plots were sampled, and the latitude and longitude coordinates of each sampled plot were measured by GPS. In each plot, the rice samples and soil samples were simultaneously collected in sample bags and soil boxes. Each sampled plot contained four subplots, we sampled 30 g of soil and a whole rice plant in each subplot. LAI was measured at each subplot with an AccuPARmodelLP-80 canopy analyzer with vertical measurement, and the average value of the four subplots was reported as the representative LAI value of the corresponding sample plot. The soil of four subplots in each sample plot were mixed for heavy metal measurement, the Cd content was determined by atomic absorption spectrometry analyzed at the Chinese Academy of Agricultural Sciences.

## 3. Methods

**Figure 2:**
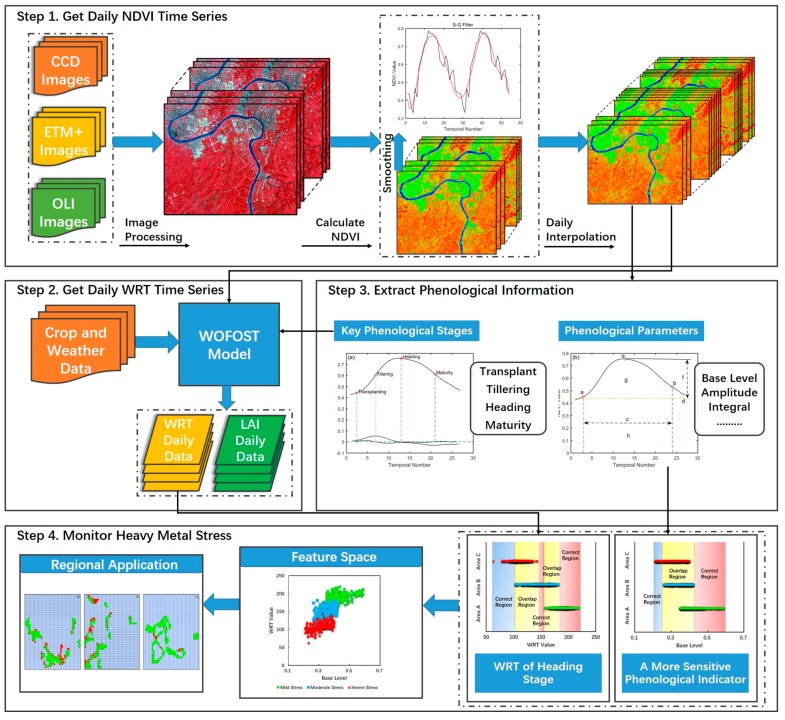
General flow chart for heavy metal stress monitoring in rice based on phenological analysis indicators and physiological parameters.

We used a comprehensive method to preprocess the remote sensing images to obtain smooth and complete daily NDVI time-series curves including image integration, NDVI calculation, denoising and daily interpolation. Then, the phenological information was extracted from the phenological cycle. The RS-WOFOST framework was applied given the difficulty in obtaining WRT data, LAI was a state variable that was acquired from both remote sensing images and the WOFOST model. A phenological indicator that was relatively sensitive to heavy metal stress in rice was chosen. Finally, a two-dimensional feature space was established based on the sensitive phenological indicator and WRT for heavy metal stress in rice. The work flow of this study is shown in Figure 2.

### 3.1. Creation of Daily Continuous NDVI Time Series

Some researchers have compared the discrepancy amongst different remote sensing images by building an intercalibration equation, and found that multi-source remote sensing images can be complementary to each other [39,40]. In view of the insufficient remote sensing images caused by the rainy season in the southern region of China, in order to collect more images to reduce the temporal interval between images, we replaced CCD images with ETM+ and OLI images when CCD images were unavailable. Thus, an integrated image set was constructed based on ETM+, OLI and CCD images which were integrated chronologically. The high consistency among images indicated the reliability of multi-source remote sensing images for time-series construction, and the non-dimensional, bounded and symmetric agreement measures are more suitable for evaluating NDVI acquired from different sensors [41]. Hence, agreement measures such as agreement coefficient (denoted as AC) and mean-square-difference (denoted as MSD) [41], were introduced for agreement analysis. The closer the AC value was to 1, the higher the consistency. MSD can be decomposed into unsystematic mean product-difference (denoted as MPDu) and systematic mean product-difference (denoted as MPDs), and the lower these three indexes, the higher the consistency:

(1) $$\mathrm{AC}\;=\;1\;-\;\frac{\sum_{i=1}^{n}{\left(\mathrm{Xi}-\mathrm{Yi}\right)}^{2}}{\sum_{i=1}^{n}\left(\left|X-Y\right|+\left|\mathrm{Xi}-X\right|\right)\left(\left|X-Y\right|+\left|\mathrm{Yi}-Y\right|\right)}$$

(2) $$\mathrm{MSD}\;=\;\frac{1}{n}\sum_{i=1}^{n}{\left(\mathrm{Xi}-\mathrm{Yi}\right)}^{2}$$

(3) $${\mathrm{MPD}}_{u}\;=\;\frac{1}{n}\sum_{i=1}^{n}\left(\left|\mathrm{Xi}-\mathrm{Zi}\right|\right)\left(\left|\mathrm{Yi}-\mathrm{Ci}\right|\right)$$

MPD_s_ = MSD − MPD_u_ (4)

where X and Y are the mean value of two image types which were evaluated, respectively; Xi and Yi are the NDVI values of each pixel, n is the number of pixels, and Zi and Ci are the predicted Xi and Yi using regression analysis.

Due to the cloud and shadow, the time-series NDVI data still had considerable noise, so that it has to be reconstructed before application [42,43,44,45]. All current methods that can be used for reconstructing do not form a unified view, which means that no method is generally applicable [42,46,47]; and problems may occur when the Gaussian filters are applied to the VI time series of irregular dilemma in CCD images [28]. In this study, we applied statistical analysis to quantitatively evaluate some commonly used denoising methods, such as Savitzky-Golay (S-G) filter, Double Logistic Fitting, and Wavelet Filter, and chose a suitable method to eliminate noise. The S-G filter is a weighted average algorithm based on a sliding window that calculates the smooth value of a fixed number of points near a certain point by the n order fitted polynomial. In the S-G filter, setting the polynomial degree and window size is vital; we obtained the best smoothing results through constant repetitions [28]. Double logistic functions are fit to data in intervals around the maxima and minima in the time series, and the local fitting function is combined to form the whole fitting function to enhance the flexibility of the fitting function and conform the fitting function to the complex behavior of the NDVI time series data [48,49]. In wavelet transform, the denoising of the time series is realized by the wavelet inverse transformation of the low frequency part and quantization of the high frequency part [50].

In the most methods that can achieve the integrity of the NDVI time series before seasonality analysis, some curve fitting methods may be too strict and would lower the efficiency of detecting the actual vegetation phenological phenomenon [42], we applied the cubic spline interpolation technology to obtain the daily NDVI time series. 

### 3.2. Derivation of Rice Phenological Characteristics

**Figure 3:**
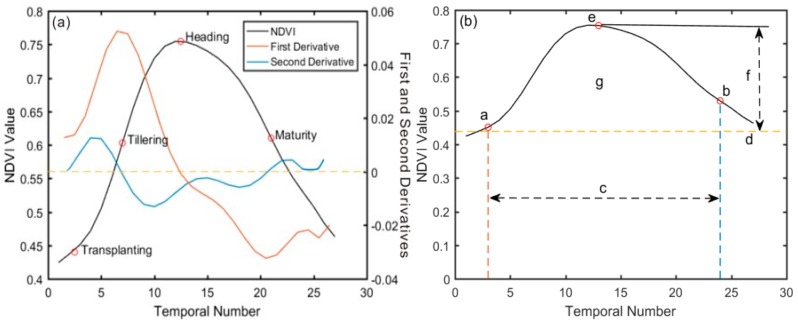
(**a**) The extraction of four key phenological stages (transplanting, tillering, heading and maturity stage) based on the first and second derivative of NDVI curve. (**b**) Application of dynamic threshold method to obtain other phenological parameters of rice. a–g represent the start of the season, the end of the season, length of the season, base level, largest value, seasonal amplitude, and seasonal integral, respectively.

We extracted the critical phenological periods and other phenological parameters using the dynamic threshold and derivation method which is based on the daily NDVI time series curve characteristics with TIMESAT [51] and MATLAB software. The biomass in the rice transplanting period was much smaller than that of the other growth stages, so judging the slight change in the NDVI curve only by using a special point was difficult. We determined the transplant period by comparing two points: the minimum point and the inflection point on the NDVI curve. The smallest NDVI value corresponded to zero of the NDVI first derivative, and the NDVI value of the inflection point was zero of the second derivative. The rice grows most rapidly at the tillering stage, so the maximum value of the first derivative indicates the active tillering stage. Due to a high level of biomass in the heading stage, we selected the maximum value of NDVI to identify this stage. At the end of the maturation stage, NDVI declined most rapidly as a result of leaf senescence, thus, the time of highest NDVI change rate (i.e., zero of the second derivative) of the curve was used to estimate the maturity stage [52]. As shown in Figure 3a, the black line corresponds to the NDVI time series curve, the other two curves are the first and second derivative curves of NDVI, respectively. In Figure 3b, a–g represents the start of the season, the end of the season, length of the season, base level, largest value, seasonal amplitude, and seasonal integral, respectively. The definitions of all the extracted seasonality parameters were displayed in Table 3. Reed et al. [17] verified that these metrics may not necessarily directly correspond to conventional, ground-based phenological events, but show strong coincidence with expected phenological characteristics. In this study, we took the length of season, base level, seasonal amplitude, growth rate, and seasonal integral as the phenological indicators for heavy metal stress monitoring, it has been proved that phenology can be a practical indicator for heavy metal stress in rice plants [15]; the significance of these parameters such as length of season, base level, seasonal amplitude, growth rate, and seasonal integral lies in the possibility to map out phenological changes in vegetation [51]. And a phenological indicator that was more sensitive to heavy metal stress in rice was chosen through intuitive comparison and pixel-based statistics of these phenological indicators.

### 3.3. Acquisition of WRT Based on Assimilation Algorithm

It is effective to apply the empirical model based on measured data and spectra to retrieve LAI with vegetation indexes (e.g., NDVI, green NDVI (GNDVI) and green-blue NDVI (GBNDVI)) [53]. In this study, several vegetation indices were investigated and we found that NDVI had a good exponential relationship with LAI measured on the ground, with a determination coefficient (R^2^) equal to 0.83. The empirical model between LAI and NDVI established in this study is shown in Equation (5):

LAI = 0.3629e^3.075NDVI^ (5)

The WOFOST model is a quantitative analysis model that simulates the distribution of roots, stems, and leaves with a time step of one day. We used data from the Zhuzhou Meteorological Station to modify the relevant crop and meteorological parameters to better describe the potential growth process of rice in the study area. In the WOFOST model, the accumulated temperature method was used to simulate the development period. Development Stage, which is equal to the ratio of actual effective accumulated temperature and needed effective accumulated temperature at each stage, describes the crop development stage. I determined the images that used for the assimilation of the RS-WOFOST framework according to extracted phenological periods, either the transplanting, tillering, heading, or maturity stage, and the Date of Emergence value was also set to adjust the phenological module in the WOFOST model, and then the assimilation of the RS-WOFOST framework was run to acquire the WRT of the whole crop growth period in MATLAB software. In addition, the stress factors were embedded in the photosynthesis positions because of the effect of heavy metal stress on photosynthesis in rice. In this study, we used the Particle Swarm Optimization (PSO) [54] algorithm to minimize the difference between observations from remote sensing and simulated values from the WOFOST model in the assimilation process with LAI as the assimilation variable, aiming at obtaining the final assimilated WRT based on the optimized parameter. The cost function is shown in Equation (6):

(6) $$Q\;=\;\sqrt{\frac{1}{N}\sum_{i=1}^{N}{\left({\mathrm{LAI}}_{R}\left(\mathrm{ti}\right)-{\mathrm{LAI}}_{S}\left(\mathrm{ti}\right)\right)}^{2}}$$

where ${\mathrm{LAI}}_{R}\left(\mathrm{ti}\right)$ and ${\mathrm{LAI}}_{s}\left(\mathrm{ti}\right)$ are the inversion LAI of the remote sensing and simulated LAI by the WOFOST model at the ith time, respectively, and N is the image number of the CCD images involved in optimization.

## 4. Results

### 4.1. Agreement Assessment of NDVI Time Series

**Figure 4:**
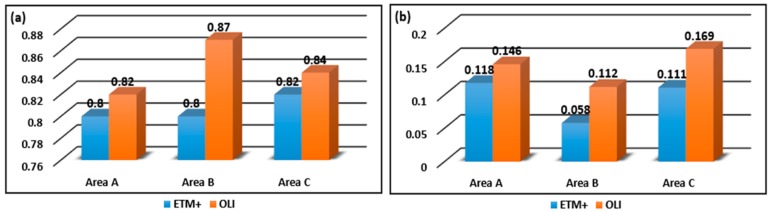

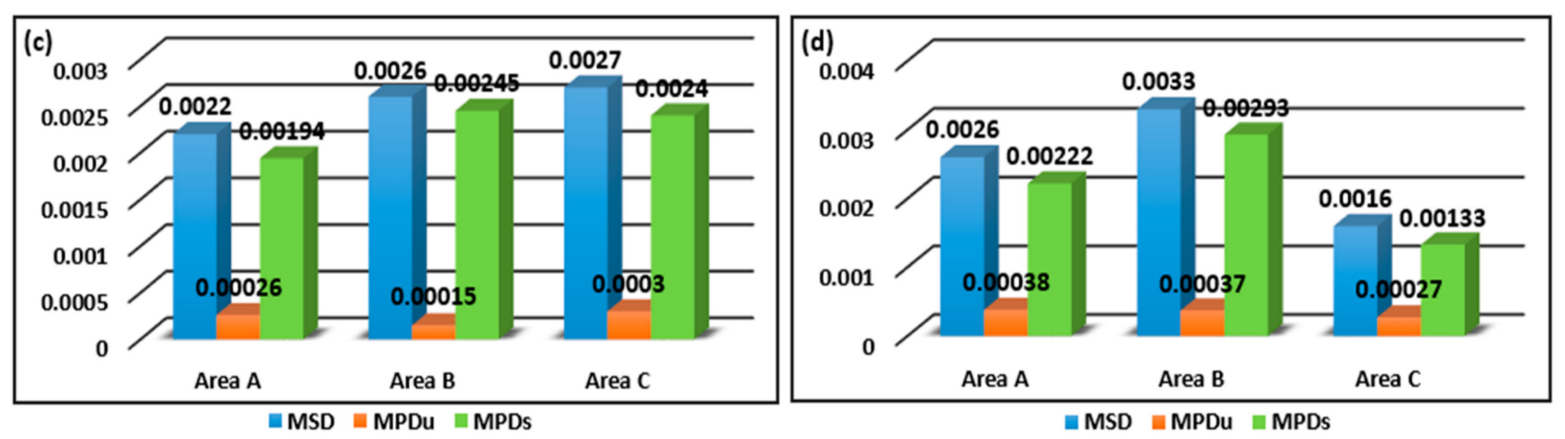
Agreement assessment of integration results: (**a**) exhibits the values of agreement coefficient (AC); (**b**) exhibits the ratio of unsystematic mean product-difference (MPDu) to systematic mean product-difference (MSD); (**c**) exhibits the values of systematic mean product-difference (MSD), unsystematic mean product-difference (MPDu) and systematic mean product-difference (MPDs) based on CCD and ETM+; and (**d**) exhibits the values based on CCD and OLI.

It is essential to carry out the agreement analysis, which is the basis for the subsequent analysis in this study. The consistency between NDVI values acquired from CCD and those obtained from ETM+ and OLI were compared by calculating the statistical analysis indicators such as AC, MSD, MPDu, MPDs, and MPDu/MSD, as shown in Figure 4. All AC values are close to 1, and the AC values based on OLI and CCD are greater than those based on ETM+ and CCD. CCD and OLI may have more consistency compared to ETM+. MSD, MPDu, MPDs, and MPDu/MSD values are all small, meaning the error among the three types of images is relatively small, verifying that integrated NDVI time series derived from CCD, ETM+, and OLI images achieved reliable results in this study. Additionally, the MPDs values are much higher than the MPDu values for the NDVI of the three datasets, indicating that systematic differences were the primary differences among the three datasets.

### 4.2. Extraction of Rice Phenology and WRT

**Figure 5:**
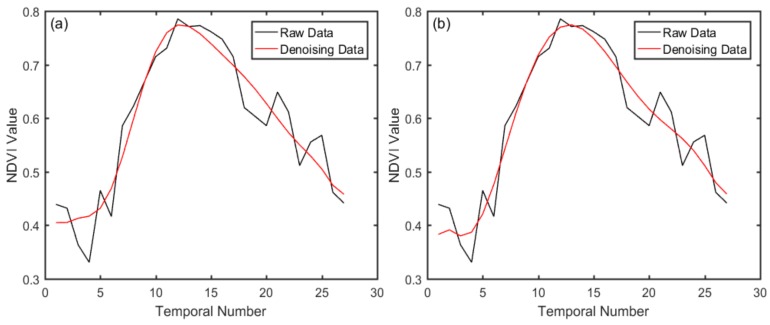

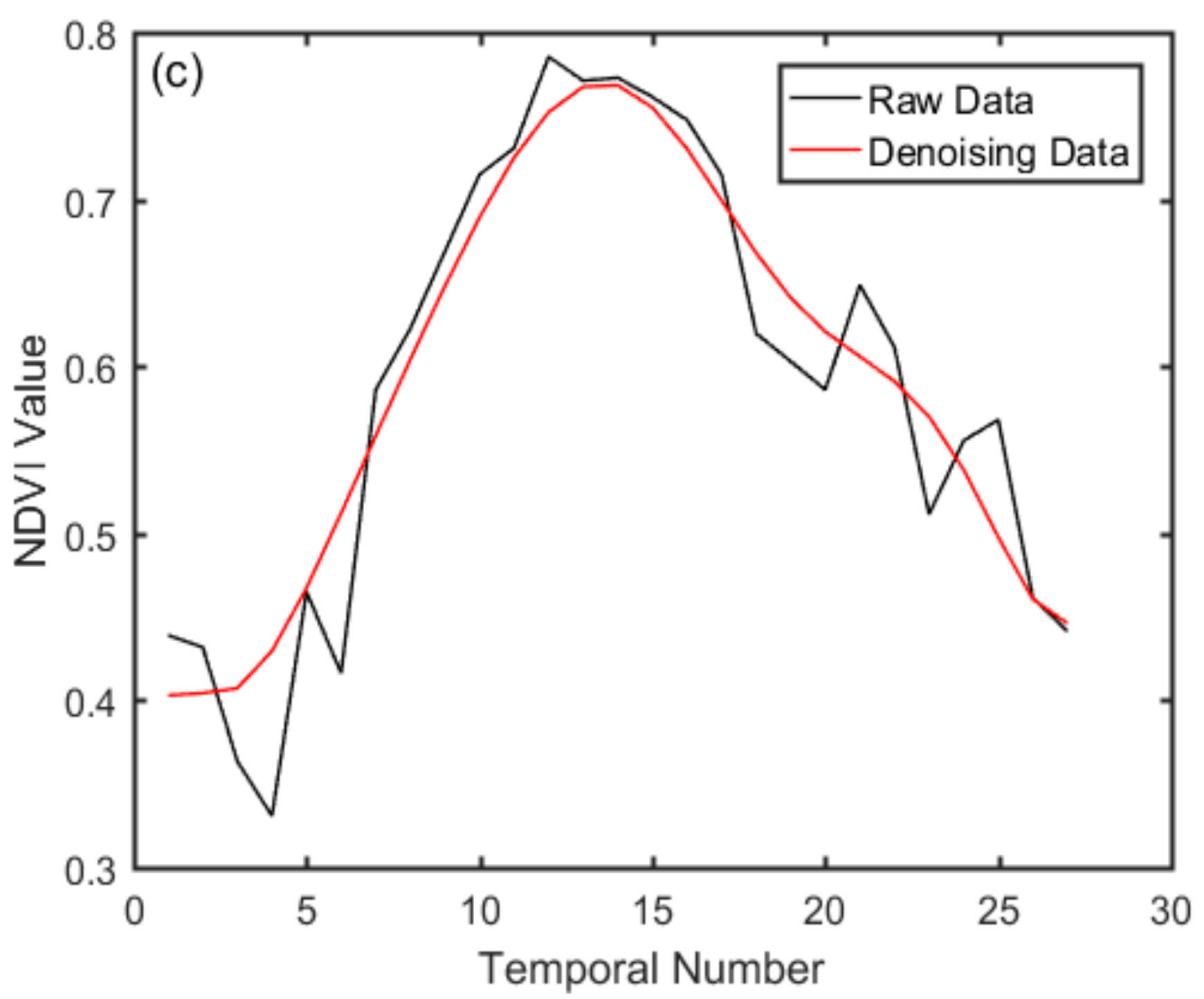
The fitting curves of three de-noising methods: (**a**) double logistic fitting, (**b**) Savitzky-Golay (S-G) filter, and (**c**) threshold wavelet transform.

From Figure 5, it can be seen that most of the noise is removed by the three methods, in comparison, the Savitzy-Golay filter is more closely related to the original data, and the curves of the double logistic function fitting and wavelet de-noising are relatively smooth. This may be due to the S-G filter emphasizing details and double logistic fitting rebuilding the overall change trend, whereas the unnecessary information was removed in the signal decomposition of wavelet transform. Statistical analysis indicators, such as mean, standard deviation, root-mean-square error (RMSE) and correlation coefficient (R), were used to further quantitatively compare the three de-noising methods. As shown in Table 4, comparing the average values of the sample data reconstructed by the three methods and the original data, the data promotion of double logistic fitting is more obvious than the other two methods. The standard deviation and root-mean-square error of S-G filter results were both the lowest among the three methods, which were 0.1171 and 0.0577, respectively. For the NDVI correlation coefficient before and after reconstruction, and the data obtained by the S-G filter had the greatest correlation with the original data with an R value of 0.8957. The S-G filter can not only remove the noise, but also fit the original data better in the curves than other methods.

**Figure 6:**
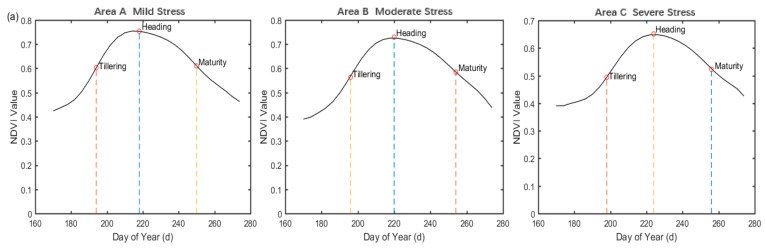

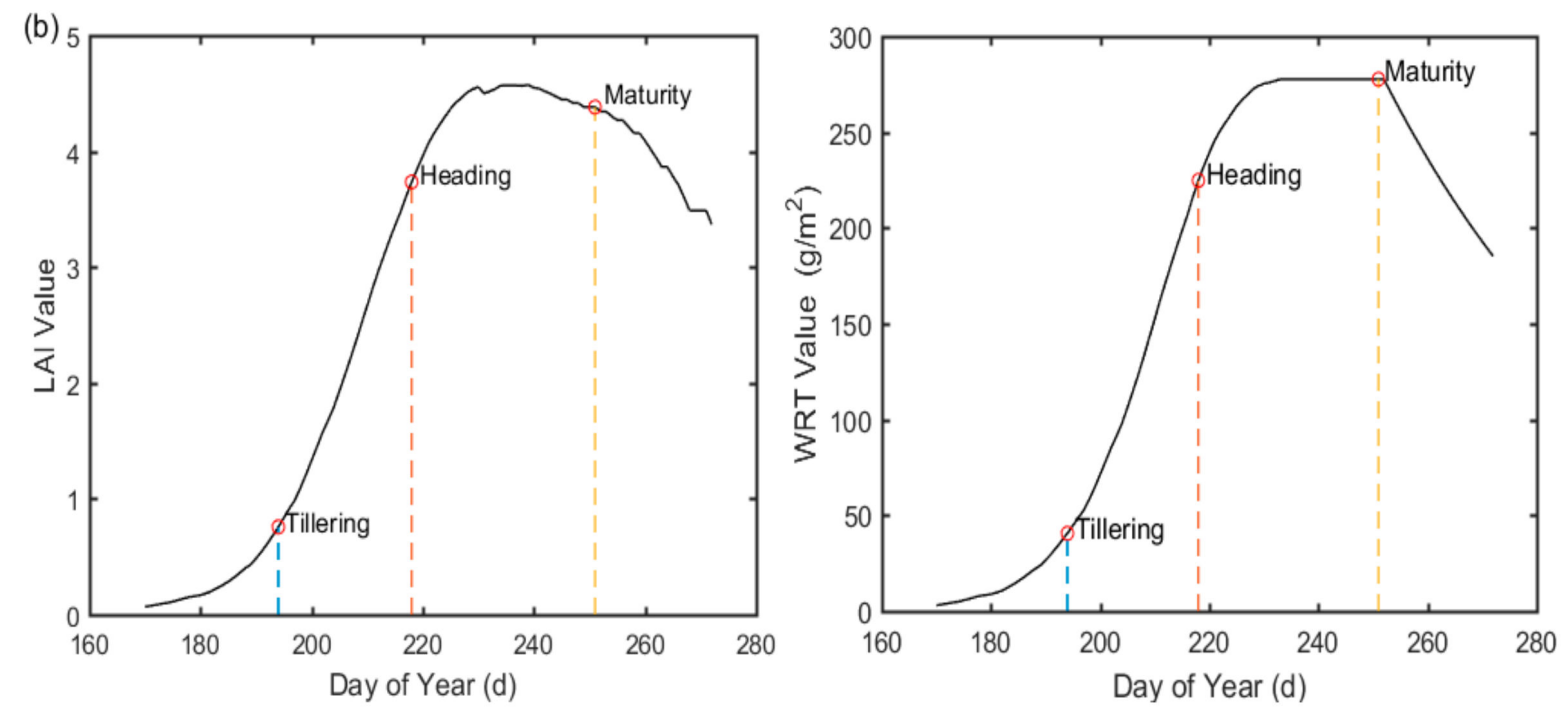
(**a**) The extraction of key phenological stages based on NDVI time series (tillering, heading, and maturity) in study areas A, B, and C respectively. (**b**) The simulation of Leaf Area Index (LAI) and Dry Weight of Roots (WRT) based on assimilation method of phenological information.

We obtained the key phenological periods for the three experimental zones based on derivative and dynamic threshold analysis. From Figure 6a, the heading date of the rice in areas A, B, and C corresponded to the 218th, 220th, and 224th day, respectively. In area A, the tillering stage was about the 194th day, and the maturity date was about the 250th day. In area B, the tillering stage occurred about the 196th day, and the maturation date was about the 254th day. In area C, the tillering was about the 198th day, and the maturity date was about the 256th day. Observing the effect of heavy metal stress on the phenology of rice only by using the difference of phenological period is insufficient, so further exploring the other phenological indicators was necessary. To adjust the WOFOST phenological module, the obtained phenological periods of rice was applied to the assimilation of the remote sensing images and the WOFOST model.

Figure 6b presents the time series of rice WRT and LAI in area A. We compared WRT and LAI curves with the daily NDVI time series. The rice grows rapidly during the tillering period, and the three curves all have considerable slope during this period. The time point corresponding to the maximum NDVI value is the heading stage. The rice enters the reproductive stage after spike differentiation, and the growth of the last leaf is completed before the end of heading stage; therefore, LAI reaches a maximum value. The rice begins to fill before the maturity stage, and first reaches the milk stage. The starch in the grains accumulates constantly and the dry and fresh weight increases, which corresponds to the section with a slight increase in the WRT curve when entering the heading period. When the rice reaches the maturity stage, the chlorophyll content decreases rapidly, the leaves gradually age and yellow, and the WRT and LAI decrease due to the competition of grain filling on the assimilates, which corresponds to the rapid decline in the two curves. At this time, NDVI declines most rapidly. The phenological stage in the LAI and WRT growth curves, corrected by phenology, is consistent with that in the NDVI time series curves.

### 4.3. Sensitivity Analysis of Different Phenological Indicators during Heavy Metal Stress

**Figure 7:**
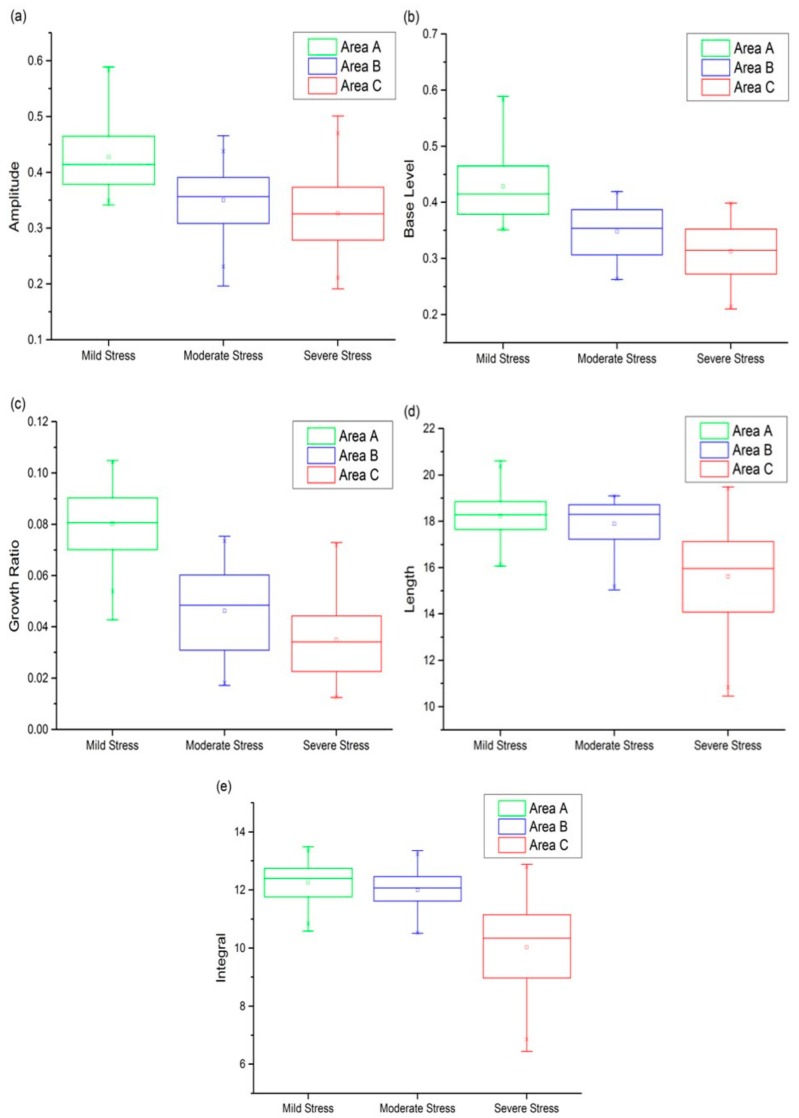
The comparison of five phenological indicators including (**a**) seasonal amplitude, (**b**) base level, (**c**) growth ratio, (**d**) length of season and (**e**) seasonal integral for analyzing heavy metal stress levels in rice.

To find out the phenological indicator that was relatively sensitive to heavy metal stress in rice, five indicators, including seasonal amplitude, base level, growth ratio, length of season, and seasonal integral were calculated in the three experimental areas, as shown in Figure 7. The statistics of phenological parameters such as average values, median, upper and lower quartiles, which corresponding to the mildly polluted areas, were generally higher than those of the moderate and severe polluted areas; that is, the heavier the heavy metal stress is, the smaller the phenological indicator values would be. According to statistical analysis, the amplitude range of areas A, B, and C were 0.34–0.59, 0.2–0.48, and 0.19–0.5, respectively. The overlap of areas B and C between the upper and lower quartiles was 69.3%, and the difference between the average values of areas B and C was 0.02427. The base level of areas A, B, and C were distributed within 0.36–0.59, 0.26–0.44, and 0.21–0.41 respectively. The overlap of areas B and C between the upper and lower quartiles was 59.6%, which was less than that displayed in Figure 7a, making the distinction between moderate and severe stress levels was more evident. It can be seen from Figure 7a,b that the base level had a higher distinguishing capability than amplitude in general. The growth rate of areas A, B, and C were in the range of 0 to 0.11. Figure 7c shows the differentiation of stress levels based on growth ratio. The overlap of areas A and B between the upper and lower quartiles was less than that based on base level, however, the overall proportion was 59%, greater than that based on base level. Compared with growth ratio, we can gain a more conspicuous discrimination result for three stress levels using base level. The trend in growth rate was consistent with previous studies that heavy metal stress reduced the growth rate of rice, which led to the unfolding of the leaves and the inhibition of radicle growth [12]. In comparison with the above phenological indicators, the differentiation in length of season was not obvious, as presented in Figure 7d. We distinguished three stress levels according to the seasonal integral (Figure 7e). 

The number of relatively high values in areas A and B was more than that in area C, which may indicate that area C was most seriously affected by heavy metal stress. However, the distribution of rice pixels in areas A and B were both concentrated in the range of 10 to 14, and are difficult to distinguish. In summary, the base level was relatively sensitive to the monitoring of heavy metal stress in the study area, whereas the length of season and the seasonal integral were not sensitive.

### 4.4. Differentiation of Heavy Metal Stress Levels Based on Feature Space

**Figure 8:**
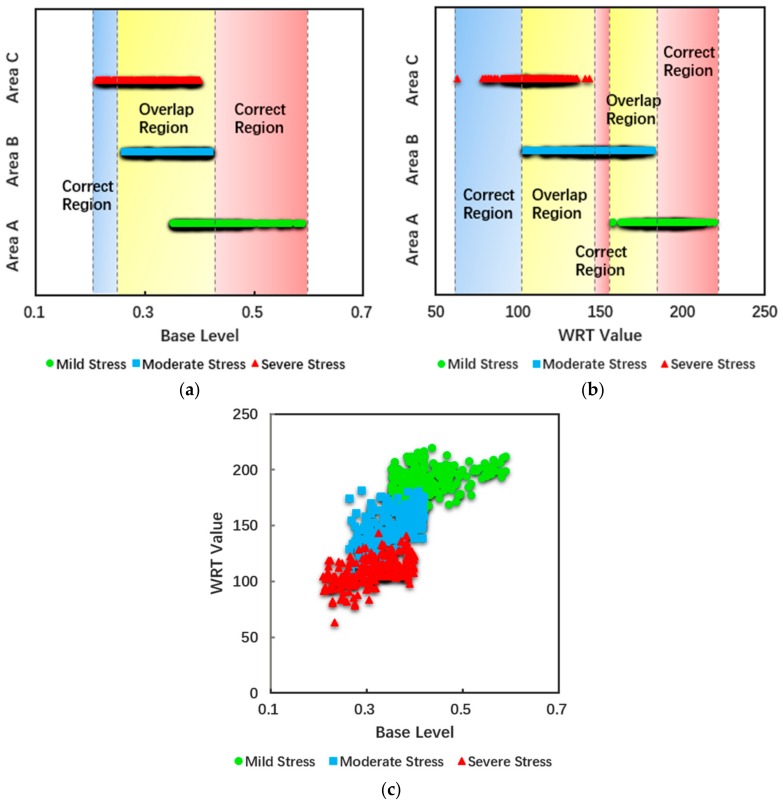
The one-dimensional judging space based on (**a**) a phenological indicator (base level) or (**b**) WRT, and (**c**) the two-dimensional judging space combining base level and WRT.

Given that the change in base level was larger than other phenological indicators in the three stress regions, the base level was selected as the most sensitive phenological indicator for this study. Although all phenological indicators were able to distinguish different heavy metal stress to a certain extent, some overlapping pixels still occurred on the divided intervals. So, we used the combination of WRT and phenology to establish a two-dimensional feature space, as taking full advantage of the two indicators can optimize stress monitoring ability. Figure 8a,b show the discrimination effect of different stress levels based on the analysis of 658 pixels for base level and WRT. The overlap area accounted for a large proportion of the total area, and had a certain effect with a single indicator for distinguishing stress level, but the accuracy was inadequate. For base level, the overlap of moderate and severe pollution areas was 70%, with mild and moderate overlapping by 50%. For WRT, the overlap of severe and moderate pollution areas was 60%, with mild and moderate overlapping by 50%. Therefore, the two-dimensional feature space was established by combining base level and WRT, as shown in Figure 8c. 

The discrimination accuracy was significantly improved, potentially indicating that the application of a two-dimensional feature space can reduce the misjudgment rate and achieve relatively accurate distinction between three different stress levels. In order to display the spatial distribution of the discriminant effect, the feature space established above was applied to the A, B, and C zones (1.28 × 1.28 km). 

**Figure 9:**
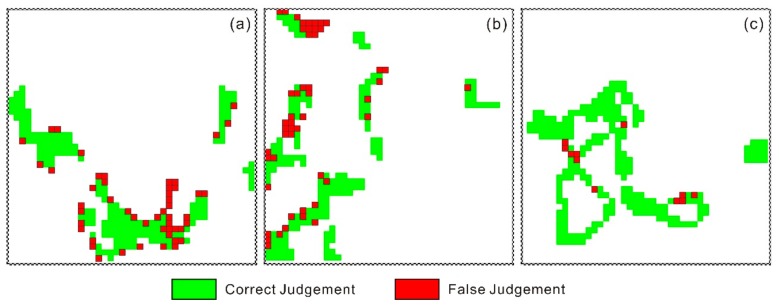
The feature space performed in (**a**) mild, (**b**) moderate, and (**c**) severe pollution areas.

Figure 9 shows the application of the feature space on the regional scale. Among them, the green part is the area that belongs to the positive judgment; the red part is the area that belongs to misjudgment. Area A had 171 pixels for positive judgment, and 50 pixels misjudged, with a positive rate of 77.4%. Area B had 159 pixels for positive judgment, and 50 pixels misjudged, with positive rate of 76.1%. Area C had 217 pixels for positive judgment, and 11 pixels misjudged, with a positive rate of 95.2%. Therefore, the feature space that combined phenology and root weight relatively effectively estimated the heavy metal stress levels in rice in the study area.

## 5. Discussion and Conclusions

Compared with other remote sensing data commonly used for monitoring vegetation phenology, CCD images have appropriate spatial and temporal resolution, which is more suitable for phenological research. The accuracy in reconstructing a NDVI time series is highly dependent on the number of cloud-free images throughout the year, so the Landsat-7 and Landsat-8 images were introduced to enlarge the number of time series images. The CCD, ETM+, and OLI Sensor specifications are generally consistent, the correlation between images based on NDVI values is high, and the consistency between the images is close to 0.9, which means we could obtain NDVI time series with smaller time interval to detect phenological information. The feasibility of multi-source remote sensing data for phenological research was verified. The performance of noise reduction and time-series construction techniques were evaluated by the ability to reflect the essential shape of the time-series, so that phenological parameters could be accurately extracted [42]. Eklundh and Jonsson suggested that the S-G filter is preferable when time series data is used to derive seasonality parameters [51]. In this study, we found that the S-G filter was superior to the other two methods in the retention of the original NDVI time series data with characteristic analysis and quantitative statistical analysis among the three common filter methods, therefore, S-G filter was chosen to reconstruct the NDVI time series curves. The NDVI time-series curves with noise deduction, were more consistent with the growth characteristics of rice.

Four key phenological periods, namely transplant, tillering, heading, and maturation, were detected through first derivative and second derivative analysis of smoothed NDVI temporal profiles. Phenological parameters such as the start of the season, the end of the season, length of the season, base level, largest value, seasonal amplitude, and seasonal integral were also extracted. TIMESAT and SPIRITS [55] were both able to extract vegetation phenology. As an exploratory study, we did not discuss the technical issue of which software is better, as it was beyond the scope of this paper. Due to the phenological indicators such as length of season, base level, seasonal amplitude, growth ratio, and seasonal integral, has important implications for the interpretation of phenological trends based on satellite time series [56]; and compared to the minor changes of physiological function or physiological factors under heavy metal stress, these metrics can reflect stress changes more effectively. Hence, these metrics were used for stress research. The results showed that the heavier the heavy metal stress is, the smaller the phenological indicator values would be, which can be used to distinguish stress levels. The results can be explained from two aspects: firstly, when the rice is under heavy metal stress, the activity of the enzyme required for chlorophyll formation is inhibited, and chlorophyll content decreased, resulting in chlorosis symptoms in rice [9,10,11,12], which performed in the NDVI time-series is the reduction of maximum and minimum NDVI values, that may reduce seasonal amplitude, base level, seasonal integral. Secondly, heavy metal stress leads to changes in rice morphology, such as curly leaves and fallen leaves. The rice cannot get enough photosynthetic products due to the reduced LAI. Meanwhile, the transport of photosynthetic products to the organs is hindered, affecting the capacity of organs to transform photosynthetic products into dry matter [31], therefore, the growth rate and the length of season may be reduced. 

We found that base level was more appropriate through the comparison of the five phenological indicators, so it was used as the sensitive phenology factor in this study. And we used the assimilation method of the WOFOST crop growth model with strong mechanism and stress factors, and remote sensing data to simulate the change in WRT, which is an effective indicator of heavy metal stress. This study presents a new method to distinguish heavy metal stress in rice, based on the feature space of phenology and WRT. Rice roots are the primary part in contact with heavy metal, so are most affected by heavy metal toxicity. After the tillering period, the susceptibility to heavy metal stress gradually decreased with the aging of the root system [57], whereas the change in photosynthesis, transpiration, and leaf area could change the phenology. The phenological changes and trends can be mapped out with phenological metrics such as base level, seasonal integral, and so on. Therefore these metrics with phenological characteristics can be used as useful indicators for monitoring heavy metal stress. As heavy metal stress changes phenology and root weight, we analyzed the mechanism of heavy metal stress and established a feature space based on the complementarity of phenology and root weight. Compared with only applying phenology or root weight, it was effective for distinguishing heavy metal stress levels through multi-dimensional characteristic space synergism.

Phenology, which refers to seasonal biological life stages driven by environmental factors, is considered to be a sensitive and accurate indicator of environmental changes [13,16], so the change in climate has considerable influence on the phenology of the rice in the study area. To eliminate the interference of the climatic changes in the research results, three experimental areas with consistent climatic changes were chosen, and the temperature, illumination, precipitation, and the comprehensive climate model were used to assess the suitability degree, which were all above 0.7, indicating that the study area was not subject to climatic conditions. Other stress factors, such as water deficit stress, nutrition, and pest stress can also cause symptoms similar to heavy metal stress. However, heavy metal stress is persistent in general, which is one important difference from other stress factors. Some studies have shown that many kinds of crops show similar symptoms to rice under heavy metal stress [3]. We can simulate the growth and development process of many annual crops all over the world by adjusting crop parameters and meteorological parameters in the WOFOST model. This study was carried out under the condition of knowing the stress factor and level beforehand. We took full advantage of phenology and WRT from the perspective of growth and stress mechanisms, and the stress levels in rice could be accurately and quickly evaluated. In addition, the phenological information and physiological characteristics attained in this study, has reference meaning for crop yield estimation and field management under heavy metal stress. It is noted that crop phenology may be subtle if the crop is under lower concentrations of heavy metals. Additionally, if this method is applied to detect heavy metal stress in rice on a large scale, it is necessary to consider different crop varieties, sowing date, plant management strategies, climatic condition, and soil composition (e.g., organic matter and PH value), which all have an impact on phenology. In the future, with a more comprehensive analysis of heavy metal stress on rice growth, the accuracy of continuous spatial-temporal evaluation of stress levels can be further improved.

## Figures and Tables

**Table 1:** The pollution levels of the heavy metal cadmium (Cd) in the three study areas.

Study Area	Geographic Location	Content of Cd in Soil (mg/kg)	Quality Standard	Pollution Level
A	113°06′E 27°47′ N	1.38	0.3–1.0	Mild
B	113°10′E 27°40′ N	2.31		Moderate
C	113°02′E 27°50′ N	3.28		Severe

Note: The materials are derived from the Hunan Institute of Geophysical and Geochemical Exploration, China.

**Table 2:** Comparison of Sensor specifications of Charged-Coupled Device (CCD), ETM+, and OLI.

Characteristic	CCD	ETM+	OLI
Spatial Resolution (m)	30	30	30
Swath Width (km)	360	185	185
Revisit Cycle (days)	2	16	16
Scan Technology	push-broom scan	push-broom scan	push-broom scan
Sensor Height (km)	649	705	705
Spectral Resolution (μm)	band1: 0.43–0.52	band1: 0.45–0.52	band1: 0.45–0.51
	band2: 0.52–0.60	band2: 0.52–0.60	band2: 0.53–0.59
	band3: 0.63–0.69	band3: 0.63–0.69	band3: 0.64–0.67
	band4: 0.76–0.90	band4: 0.76–0.90	band4: 0.85–0.88

**Table 3:** Satellite-derived phenological NDVI metrics presented in this study.

Variable	Definition	Reported Unit
Start of Season	Starting point of the growing season	Day of year
End of Season	Ending point of the growing season	Day of year
Length of Season	Interval elapsed from the start to the end of the season	Number of days
Base Level	Average of the left and right minimum values	NDVI unit
Largest Value	Highest value of a year	NDVI unit
Seasonal Amplitude	Difference between the maximum value and the base level	NDVI unit
Seasonal Integral	Integral of the function describing the season from season start to season end	(NDVI unit)·(time unit)
Rate of Increase or Decrease	Ratio of the difference between the left or right 20% and 80% levels and the corresponding time difference	(NDVI unit)/(time unit)

**Table 4:** The comparison of three de-noising methods using average value, standard deviation, root mean square error (RMSE), and correlation coefficient.

	Original	Double Logistic Fitting	Savitzky-Golay Filter	Threshold Wavelet	Forced Wavelet
average value	0.5820	0.5946	0.5925	0.5921	0.5919
standard deviation	0.1351	0.1178	0.1171	0.1205	0.1204
root mean square error	/	0.0624	0.0577	0.0620	0.0649
correlation coefficient	/	0.8752	0.8957	0.8775	0.8636
